# Measuring Effects of Metaphor in a Dynamic Opinion Landscape

**DOI:** 10.1371/journal.pone.0133939

**Published:** 2015-07-28

**Authors:** Paul H. Thibodeau, Lera Boroditsky

**Affiliations:** 1 Department of Psychology, Oberlin College, Oberlin, Ohio, United States of America; 2 Department of Cognitive Science, University of California, San Diego, La Jolla, California, United States of America; IFIMAR, UNMdP-CONICET, ARGENTINA

## Abstract

Metaphors pervade discussions of critical issues, making up as much as 10–20% of natural discourse. Recent work has suggested that these conventional and systematic metaphors influence the way people reason about the issues they describe. For instance, previous work has found that people were more likely to want to fight back against a crime *beast* by increasing the police force but more likely to want to diagnose and treat a crime *virus* through social reform. Here, we report the results of three norming tasks and two experiments that reveal a shift in the overall landscape of opinion on the topic of crime. Importantly, we find that the metaphors continue to have an influence on people’s reasoning about crime. Our results and analyses highlight the importance of up-to-date opinion norms and carefully controlled materials in metaphor research.

## Introduction

Metaphors pervade discussions of critical issues, making up as much as 10–20% of natural discourse [1]. Many metaphors are conventional and systematic [2]. For example, we commonly use terms that are associated with disease to talk about crime, as in “Crime *plagues* and *infects* cities.” Further, most complex social issues are talked about using more than one system of metaphors. For example, in addition to disease metaphors we can also talk about crime as a beast or wild animal, as in “Crime *attacks* and *preys on* cities.”

Recent work [3–6] has suggested that these metaphors are more than simply colorful ways of talking. Using different metaphors leads people to reason differently about social issues and to follow different paths of inference. For example, when people read a report that described crime as a *beast*, they were more likely to want to *fight back* by increasing the police force. When they read that crime was a *virus* they preferred *diagnosing* and *treating* the problem through social reform [5–6].

Of course, metaphors about social issues exist in a dynamic public discourse. As topics gain and lose popularity, get reframed in the news cycle, and are reconsidered in light of new events, public opinion and public engagement on different issues can shift. In this paper, we revisit two earlier sets of studies (conducted from 2008 to 2011, [5–6]), replicate the results and compare our new data with both of the older data sets and with other recently collected samples [7].

### Plan of the Paper

This paper follows up on the findings reported in Thibodeau and Boroditsky [5–6]. The original studies demonstrated that metaphorically framing crime as a *beast* led people to seek more enforcement-oriented policies (harsher prison sentences, more police patrols) while framing crime as a *virus* led people to prefer reform-oriented solutions (improving economic welfare and reforming education). The same pattern was replicated across 6 experiments, whether participants generated their own solutions or picked among alternatives, and across several other variations in methodology and materials [5–6].

Here we present a new set of follow-up studies and offer a detailed analysis of the data. Rather than simply looking at a reduced dichotomous coding of responses, we consider the full pattern of participants’ choices. We also present a new set of norming studies, run in the same time-period as the new replication studies. These norms provide a necessary measure of participants’ understanding of the response choices against which the data can be interpreted. Further, we reanalyze data from a study published by another laboratory [7] that also show a metaphor framing effect, when current attitudes toward crime are taken into account.

We find that the overall choice pattern, and critically, participants’ interpretation of one of the response options, has shifted since 2011. In norms conducted in 2011 the suggestion of reducing crime by developing “neighborhood watch” programs was seen as more enforcement-oriented and consistent with the *beast* frame, whereas in norms conducted in 2014, participants viewed this same response option as more reform-oriented and consistent with the *virus* frame. When this response option is coded appropriately with respect to the relevant opinion norms, we see the same effect of metaphorical frames as reported in [5–6].

Next, to make sure that the effects of metaphorical framing do not depend on the interpretation of this particular response option, we conduct a study that excludes this policy response. We again replicate the original effects of metaphorical frame.

Finally, we consider potential non-metaphorical versions of our stimuli, framing crime as a *problem* or *horrific problem* instead of as a *virus* or a *beast*. We present the results of a norming study that highlights the complexity and nuance of linguistic stimuli. While it is tempting to imagine non-metaphorical “equivalents” for metaphorical frames as control stimuli, we show that such a substitution introduces an assemblage of uncontrolled changes in meaning, vividness, and arousal.

We conclude with a discussion of methodology practices and challenges in modern research on language and reasoning, and address some frequently asked questions, including the implications for the burgeoning culture of rapid replication in the behavioral sciences.

## Methods

The analyses described in this paper include data from three sources, including samples reported in Thibodeau and Boroditksy [5–6] and by Steen, Reijnierse, and Burgers [7]. Data previously reported in [5–6] are available at https://osf.io/r8mac/. Data from [7] were downloaded from https://osf.io/ujv2f/. In addition, we collected data for two novel experiments and three norming tasks, which are also available at https://osf.io/r8mac/, along with analysis files. The samples and methods for the novel studies are described in this section.

### Ethics Statement

The experiments reported here were done in accordance with the Declaration of Helsinki. Additionally, they followed the ethical requirements of the Oberlin College institutional review board and complied with ethics guidelines set forth by the IRB recommendations; the Oberlin College institutional review board reviewed and approved the protocol for studies presented here. Participants were informed that their data would be treated anonymously and that they could terminate the experiment at any time without providing any reason. We received informed consent from all participants before they participated in an experiment. The first page of the study described the potential risks and benefits of participation. Upon agreeing to these conditions, participants clicked a radio button as an indication of their consent; they were then provided with additional instructions and the experimental materials.

### Participants

Four samples of participants were collected for the present analyses. Each sample was recruited from Amazon’s Mechanical Turk (mturk.com) using three exclusion criteria: we restricted our sample to participants living in the United States, who were at least 18 years old, with a performance rating of at least 90%. We paid people $1.00 in exchange for their participation.

The surveys were implemented in Qualtrics. At the end of each study, participants were given a completion code, which they were instructed to submit into the Turk interface. Data from participants who failed submit an accurate completion code were excluded from analyses.

Since variants of this experiment have been run on Mechanical Turk several times (e.g., [5–7]), we asked participants in Experiments 1 and 2 if they had ever completed a similar study at the end of the survey: “Have you previously completed a study that is closely related to this one in which you read a story about crime and had to make a decision about what kind of response the community should adopt?” Participants who indicated that they had completed a similar study were excluded from analyses.

After completing the study, participants were asked background questions, including their age, sex, political affiliation, and educational history (see Table 1 for full demographic information by study).

**Table 1:** Participant information for the norming studies and experiments.

	Norming 1:Responses	Norming 2:Frames	Experiment 1:Five options	Experiment 2: Two options
Sampled	250	250	650	650
Analyzed N	242	248	526	521
Age: *M* (*sd)*	32.3 (11.4)	32.7 (10.8)	32.2 (10.3)	32.6 (10.9)
Male	111 (46%)	144 (58%)	296 (56%)	278 (53%)
Democrat	81 (34%)	99 (40%)	200 (38%)	213 (41%)
Independent	93 (38%)	103 (42%)	241 (46%)	226 (43%)
Republican	31 (13%)	46 (19%)	85 (16%)	82 (16%)
Education: Some college	205 (85%)	221 (89%)	460 (87%)	459 (88%)

The five-response-option version of this experiment in previous work yielded effect sizes of .17 and .15 (Cohen’s *w*) [6]. A power analysis (*β* = .8) revealed that, given this effect size, our sample should include between 150 and 200 participants in each of the two metaphor framing conditions. We estimated that as many as 25% of the participants that we sampled would have to be excluded because they had previously participated in a related study or because they would submit an incorrect completion code. As a result we collected data from 650 participants in Experiments 1 and 2 (in which there were two framing conditions).

Data from eight (3.2%) and two (0.8%) participants were excluded from the first and second norming studies because an incorrect completion code was submitted. Data from 84 (12.9%) and 98 (15.1%) participants were excluded from Experiments 1 and 2 because an incorrect completion code was submitted or because participants reported having completed a similar study (e.g., [5–7] or one of the norming studies).

We also excluded data from participants who reported that English was not their first language and from participants who reported that they did not live in the US. This included 14 (2.5%) and 15 (2.7%) participants from Experiments 1 and 2, respectively. In line with [7], we also excluded data from participants who responded extremely quickly or slowly (i.e. faster than 10 seconds or slower than 300 seconds). This led to the exclusion of data from 26 (4.7%) and 16 (3.0%) participants in Experiments 1 and 2 respectively.

There were no differences across conditions by age, sex, political ideology, or education in the two experiments.

### Materials

#### Norming study 1 (norming tasks 1 and 2)

In the first norming study, participants were asked to complete two tasks. First, they were asked to match policy approaches to metaphors. After reading a non-metaphorically framed version of the original crime report, they were asked to match one of the five response options to the *beast* metaphor and one to the *virus* metaphor. The complete text for this task read:

Addison is a city with a crime problem. Five years ago Addison was in good shape, with no obvious vulnerabilities. Unfortunately, in the past five years the city's defense systems have weakened, and the city has succumbed to crime. Today, there are more than 55,000 criminal incidents a year—up by more than 10,000 per year. There is a worry that if the city does not regain its strength soon, even more serious problems may start to develop.

The city's officials know that they have to change certain policies in response to the problem, but they aren't sure which policies to change or how much to change them. Two of the city's officials are leading this debate and they tend to talk about the problem in different ways.

One argues that "Crime is a VIRUS ravaging the city of Addison."

The other argues that "Crime is a BEAST ravaging the city of Addison."

If you had to guess, which of the crime-reducing options listed under the "Items" menu is supported by each of the officials. Pick item that you think is most consistent with the virus expression, then drag and drop that item into the "Virus" box. Do the same with the proposal that is most consistent with the beast expression and drag and drop it into the "Beast" box (1 in each).

The five policy responses read:

1. Increase street patrols that look for criminals.
2. Increase prison sentences for convicted offenders.
3. Reform education practices and create after school programs.
4. Expand economic welfare programs and create jobs.
5. Develop neighborhood watch programs and do more community outreach.

In the second task participants were asked to rate the degree to which each of the five policy approaches emphasized enforcement versus reform using a 101-point scale (-50 = completely reform-oriented; 50 = completely enforcement-oriented; this scale was subsequently adjusted to range from 0 to 100). The instructions for this task read:

The following approaches to reducing crime vary in the degree to which they emphasize law enforcement and social reform. For each of the policy approaches below, please rate the extent to which, in your view, each option emphasizes law enforcement versus social reform.

Use the slidebar to indicate your rating of each of the five policy approaches. More extreme negative values indicate a view that the policy approach is relatively reform-oriented; more extreme positive values indicate a view that the policy approach is relatively enforcement-oriented.

The order of the policy options in both of the norming tasks was randomized. The matching task always preceded the rating task.

#### Norming study 2 (norming task 3)

In the second norming study participants were asked to rate four frames along a variety of dimensions. The frames read:

1. Crime is a beast ravaging the city.
2. Crime is a virus ravaging the city.
3. Crime is a problem ravaging the city.
4. Crime is a horrific problem ravaging the city.

Participants rank ordered the four frames in terms of how “severe they make the issue of crime seem.” Then they rated the severity, metaphoricity, and conventionality of the four frames (on 101-point scales): “Please rate these descriptions of crime in terms of how {severe they make the issue seem, metaphorical the language is, common or conventional the language is}.” Finally, participants were asked to select “which of the four descriptions [they] would be most likely to use to describe a severe crime problem.” These questions were asked on separate screens.

#### Experiment 1

In Experiment 1 participants were randomly assigned to one of two framing conditions. In one, crime was described as a *virus* ravaging the city; in the other crime was described as a *beast* ravaging the city. The remainder of the report (i.e. the original version used in [6] and [7]) was identical across conditions. After reading the report participants were asked to rank order the five response options. The amount of time that participants spent reading and ranking the responses was recorded.

On the subsequent screen participants in each of the three experiments were asked to recall the frame that they had read during the task: “The crime report that you read began by saying ‘Crime is a _________ ravaging the city of Addison.’ Please fill in the blank to the best of your ability from memory (do not click the back button to check the story).” Unfortunately, due to a coding error data from 135 (25.7%) participants did not receive this question. Data from these participants were excluded from one set of analyses (a test of whether the metaphors covertly affected reasoning).

Finally, in both experiments participants were asked a set of background demographic questions, including their age, sex, educational background, first language, geographic location, and political affiliation.

#### Experiment 2

The materials and design for Experiment 2 were identical to those of Experiment 1, except that participants were asked to choose between two policy responses:

1. Increase street patrols that look for criminals
2. Reform educational practices and create after school programs

As with Experiment 1, a coding error led to 135 (25.7%) participants not receiving the follow-up cued recall question.

### Coding

#### Ratings

Analyses in prior work [5–7] coded the policy responses into two categories: those that were enforcement-oriented and those that were reform-oriented. To establish an empirical basis for this coding, we collected ratings of the five policy approaches along a dimension of enforcement versus reform [6]. In that study, we found that the “patrols” (*M* = 87.21, *sd* = 13.5), “prison” (*M* = 85.11, *sd* = 22.37), and “neighborhood watch” (*M* = 58.69, s*d* = 25.77) options were viewed as enforcement-oriented (i.e. above the midpoint, 50, of a scale that ranged from 0, very reform oriented to 100, very enforcement-oriented). The other two response options–“education” (*M* = 17.14, *sd* = 27.13) and “economy” (*M* = 20.82, *sd* = 30.93)–were, on the other hand, viewed as reform-oriented. However, we noted that the “neighborhood watch” “option was not rated as extreme as ‘street patrols’ or ‘prison sentences’ suggest[ing] that it may represent a more balanced approach. For this reason, we did not include this option in the response set in Experiments 1 or 2” (p. 4 of [6]).

We repeated this norming task for the present paper to test whether people maintained a consistent view of these policy approaches over this period of time. There are several important differences in the sociopolitical context today (i.e. in 2014) relative to when the initial studies were conducted (i.e. between 2008 and 2011) that may make people think differently about crime in general or these policy approaches in particular. For instance, an economic crisis was at its peak when the initial samples were collected. In addition, the initial studies pre-dated salient incidents related to race, policing, and social justice that were major news issues in 2013 and 2014.

We found that the options that had been rated as strongly enforcement-oriented in the past were rated as strongly enforcement-oriented in the current sample: “patrols” (*M* = 86.901, *sd* = 14.965), and “prison” (*M* = 87.479, *sd* = 18.351). We also found that the options that had been rated as strongly reform-oriented in the past were rated as strongly reform-oriented in the current sample: “economy” (*M* = 14.752, *sd* = 21.522) and “education” (*M* = 15.372, *sd* = 21.578).

**Fig 1:**
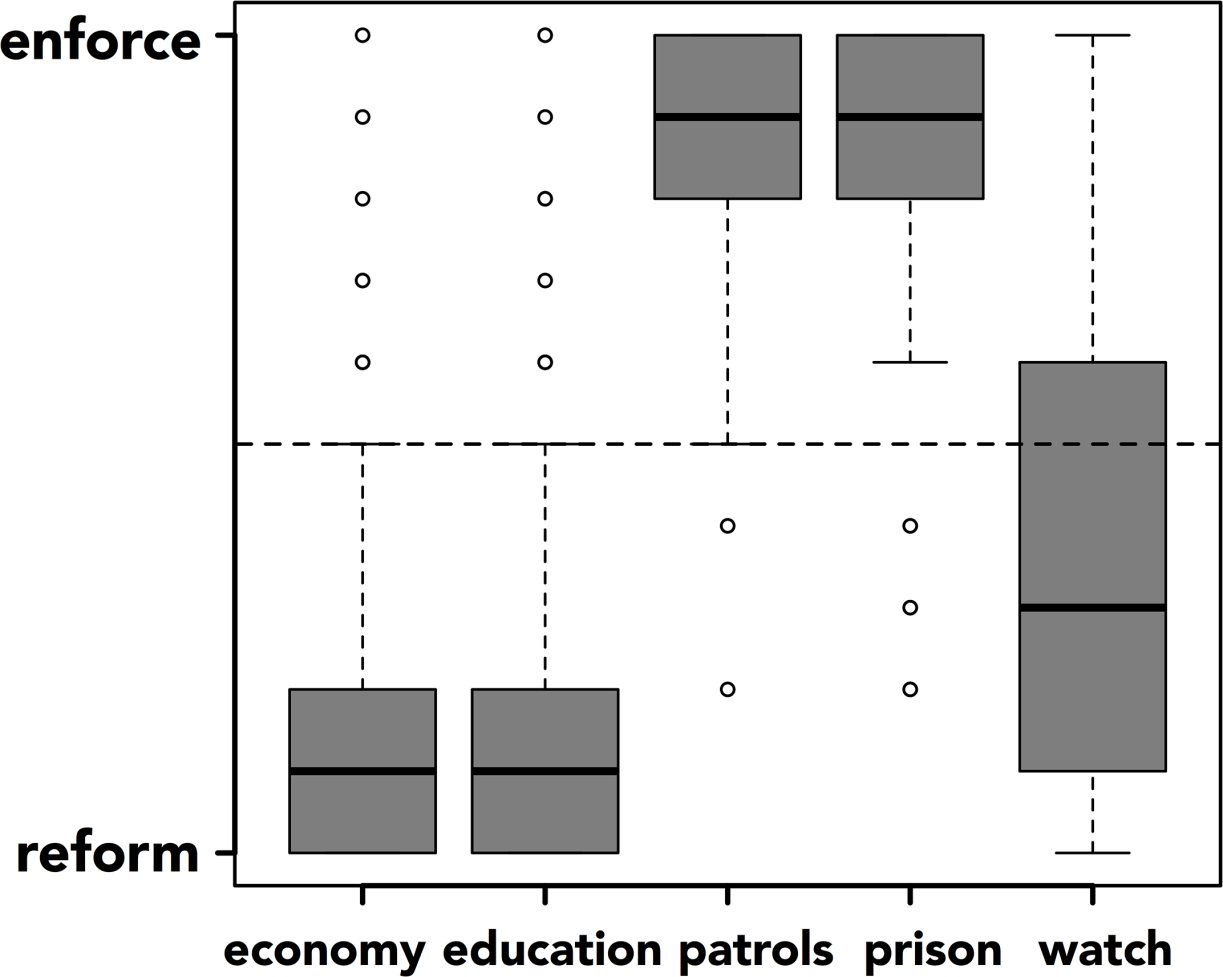
Ratings of the five policy responses along a dimension of reform- to enforcement-oriented.

The “neighborhood watch” option, however, was rated as more reform-oriented (*M* = 36.736, *sd* = 28.556) in the current study, significantly below the midpoint of the scale, *t*[241] = 7.226, *p* < .001, and significantly different from how it was rated in earlier studies, *t*[46.936; variances not assumed to be equal; n_1_ = 35, n_2_ = 242] = 4.643, *p* < .001 (see Fig 1).

Note that ratings for the other four policy options did not change significantly over this time: “patrols”, *t*[43.38] = .12, *p* = .903; “prison”, *t*[40.89] = .60, *p* = .554; “education”, *t*[40.46] = .37, *p* = .714; “economy”, *t*[38.90] = 1.12, *p* = .268.

### Matching

Although the distinction between enforcement and reform is an important one, it may not be the best way to think about the relationship between the metaphors and policies. As we have argued, our theory is that the frames will make people more likely to select policies that are congruent with the entailments of the metaphors.

**Fig 2:**
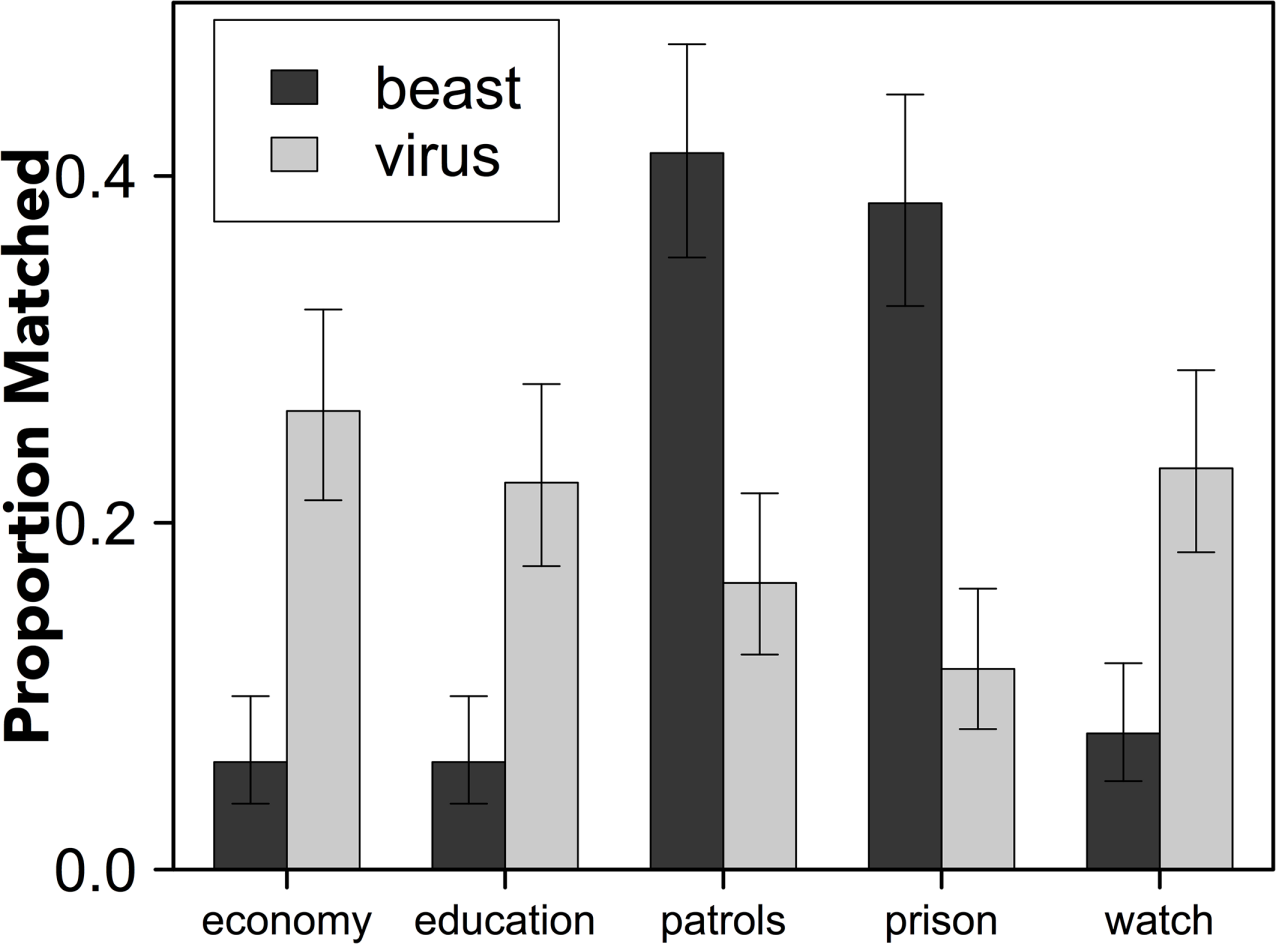
The proportion of policy responses that were matched to the *beast* and *virus* metaphors. Error bars reflect 95% confidence intervals.

To gauge which policy approaches were viewed as consistent with each of the frames, we asked participants to complete a second norming task (with the same sample that completed the first norming task). We presented participants with the original crime report (without a metaphor frame) and indicated that different politicians were using different metaphors to support different policies. We asked participants to match one of the five policies to the *beast* metaphor and one to the *virus* metaphor. Note that a similar norming task was reported in [6]; however, the “neighborhood watch” option was not included in the response set because we had not included it as a response option in earlier studies [5] or Experiment 2 of [6]. Fig 2 illustrates how participants viewed the relationships between the policies and metaphors in the current study.

Separate chi-square goodness of fit tests revealed that people matched the policies unevenly to the *beast* frame, *χ* ^*2*^(4, N = 242) = 160.066, *p* < .001, and to the *virus* frame, *χ* ^*2*^(1, N = 242) = 16.926, *p* = .002. That is, there was consensus among the sample that certain policies seemed to fit better with the *beast* frame than others and that certain policies seemed to fit better with the *virus* frame than others.

To determine which policies were viewed as fitting best with each metaphor we conducted five separate repeated measure logistic regressions (Bonferroni-corrected α = .01)–one for each policy response. This analysis revealed that participants were more likely to match the “economy”, *χ* ^*2*^(1) = 38.666, *p* < .001 (*β* = -1.694, *SE* = .304, *p* < .001), “education”, *χ* ^*2*^(1) = 27.07, *p* < .001 (*β* = -1.469, *SE* = .308, *p* < .001), and “neighborhood watch”, *χ* ^*2*^(1) = 22.429, *p* < .001 (*β* = -1.262, *SE* = .284, *p* < .001) policies to the *virus* frame; participants were more likely to match policies that emphasized “patrols”, *χ* ^*2*^(1) = 37.085, *p* < .001 (*β* = 1.269, *SE* = .217, *p* < .001), and “prison” sentences, *χ* ^*2*^(1) = 48.529, *p* < .001 (*β* = 1.562, *SE* = .241, *p* < .001) to the *beast* frame.

This analysis suggests that policies that address the economy, educational system, and neighborhood watches are viewed not only as more reform-oriented but should be coded as congruent with the *virus* frame. In contrast, policies that emphasize increasing police patrols and extending prison sentences should be coded as congruent with the *beast* frame.

One interesting detail of this effect is that the pattern was cleaner for the *beast* metaphor than *virus*. This may be because the entailments of the *virus* frame are less clear than the entailments of the *beast* frame, as people often talk about *fighting* viruses, infections, and disease (e.g., [3]).

In several of the analyses below, we will report the results of two methods for coding the policies: one that is consistent with the older set of norms [6], in which the “neighborhood watch” option is categorized as consistent with the *beast* metaphor; and one in which the response options are coded in a way that is consistent with the results of the more recent norming study, in which the neighborhood watch option is categorized as consistent with the *virus* metaphor.

### Reanalyzing Previously Published Data

In addition to the new data collected in Experiment 1, we also analyze data from a very similar study conducted by another laboratory in 2014 [7]. Here we focus on the results of Experiment 4, which was the only study with a sufficiently large sample to be able to detect an effect of a metaphorical frame. Also, because the data in this sample were collected close in time to our new sample and norming studies (all data collected between August 2014 and December 2014), it is the only study from the set for which we have relevant norming data.

Note that Steen et al [7] conducted four experiments to test for framing effects on crime. Experiment 1 was conducted in Dutch while Experiments 2–4 were conducted in English. In every case there were six framing conditions in a 2 (metaphor support: present or absent) by 3 (frame: *virus*, *beast*, or *problem*) between-subjects design. As the authors acknowledge, the sample sizes in Experiments 1, 2, and 3 were underpowered (with as few as 36 participants in a cell). This is far fewer than what is called for given the effect size. Indeed, even Experiment 4, which included a substantially larger sample, may be underpowered (with as few as 162 participants; as many as 180), since the replication conditions were predicted to yield the largest differences. Adding novel conditions for which intermediate effects are predicted requires even larger samples of data.

Further, although the authors of [7] analyzed the effects of the frames on participants’ top-ranked policy preference in secondary analyses, the primary analyses were conducted on data in which the top-two policy preferences were aggregated: “We included the first two preferences for the five policy measures that were rank-ordered by participants, coding reform measures as 0 and enforcement measures as +1. This yields a scale with three values: each participant either preferred two enforcement-oriented measures (+2), one enforcement-oriented and one reform-oriented measure (+1) or two reformed-oriented measures (0)” (p. 13). The authors fit linear ANOVA models to these data.

This approach is inconsistent with that of [5–6], and is problematic for psychological and statistical reasons [8]. On psychological grounds, it is likely that participants most carefully considered their top ranked option. On statistical grounds, it is more appropriate to treat these data (either the top choice or an aggregate measure of the top two choices) as categorical [8]. Although it is common to analyze categorical data using ANOVAs, this approach “*can lead to spurious null results and spurious significances* … [that] go beyond the normal chance of Type 1 and Type II errors” [p. 435, 8, italics in original]. For instance, in considering participants’ top two policy preferences, there are only three levels to the scale, but probability mass is assigned to values that can never occur (i.e. values less than 0 or greater than 2). For these reasons (i.e. consistency with previous work and statistical appropriateness), we will analyze participants’ top choice using statistical techniques for categorical data analysis (i.e. logistic regression and chi-square).

### Modeling Data

Many of the analyses that we report involve fitting logistic regression models with several predictors. The primary benefit of fitting logistic regression models is that we can include demographic characteristics as covariates. This is important as we have found that people with, for instance, different political ideologies tend to think differently about crime and are affected differently by the frames [5–6]. To find the best fitting logistic regression models for the data, we utilized a stepwise model selection algorithm from the MASS library in *R* [9–10]. This algorithm takes a maximally parameterized model and tests alternatives that include subsets of predictor variables by comparing AIC values (by both pairing down from the maximally parameterized one and working up from the minimally parameterized one) in order to find the best fit for the data [11].

For consistency, we fit the same maximally parameterized model in every analysis. This model included tests of main effects by frame (e.g., *beast*, *virus*), time spent reading the report, political affiliation (Democrat, Republican, Independent), age, sex, and education. This model also included tests for interactions between the frame and covariates (e.g., the frame and time spent reading the report; the frame and political ideology; the frame and age; etc). As a result, in most cases the initial models included 14 parameters. We report the best fitting model as well as indices of fit (AIC) for the best fitting model, the maximally parameterized model, and the minimally parameterized model (i.e., one that solely included an intercept).

## Results

### Do the “virus” and “beast” metaphors affect the way people think about crime?

We first present analyses based on the full distribution of responses. We then analyze the data as dichotomously coded according to old opinion norms (collected in 2011), and new opinion norms (collected in 2014).

### The Full Distribution of Responses

Before looking at the effect of the frames on responses, we examined the overall popularity of each policy by experiment. We found that in both Experiment 4 of [7] and our Experiment 1, participants were extremely unlikely to choose the option having to do with the “prison system” (3.4% and 7.4%). In [7] the “neighborhood watch” option was the most popular (30.9% selected) whereas in our study, the option calling for increased “street patrols” was the most popular (29.5%). Despite this similarity, we found a difference in the distributions of policy preferences across the two experiments, *χ* ^*2*^(4, N = 876) = 24.554, *p* < .001 (see Table 2).

**Table 2:** The frequency (and proportion) of each policy chosen in Steen et al.’s Experiment 4 [7] and the current Experiment 1.

	Economy	Education	Patrols	Prison	Watches
Experiment 4	91 (26.0%)	67 (19.1%)	72 (20.6%)	12 (3.4%)	108 (30.19)
Experiment 1	91 (17.3%)	120 (22.8%)	155 (29.5%)	39 (7.4%)	121 (23.0%)

One possible explanation for this difference may have to do with the ordering of the policy options in the two experiments. In our prior work [5–6] and in Experiment 1, we randomized the order in which the policy approaches were presented to participants. It is not clear whether this was done in Experiment 4 of [7] as the authors do not report that the order of the policies was randomized. In Experiment 1, we found that participants were more likely to select options presented first or last compared to options that were presented in the middle, χ^2^(1, N = 526) = 8.791, *p* = .003. This may lead to overall differences between the policy preferences by sample, but it is unlikely to have affected the relationship between the frames and policy preferences (i.e. it is unlikely that randomizing–or not randomizing–the order of policies would affect people who read that crime was a *beast* differently than people who read that crime was a *virus*).

**Fig 3:**
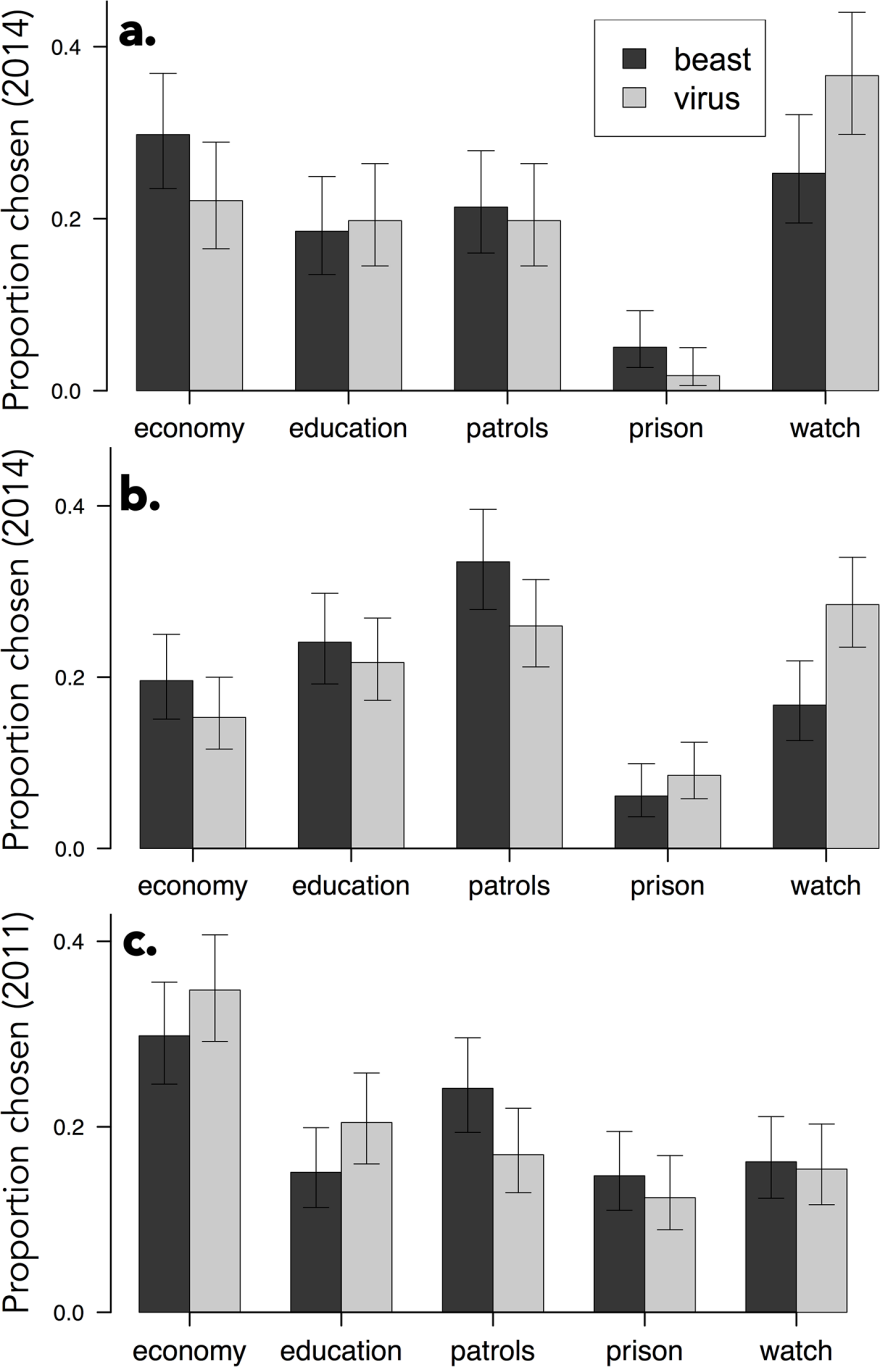
The distribution of policy choices by metaphor frame for three samples. a. Steen et al.’s Experiment 4 of [7] (from the *virus* and *beast* original report conditions); b. the current Experiment 1; and c. data pooled from Experiments 3 and 4 of [6]. Error bars denote 95% confidence intervals.

We next tested for the effects of the metaphor frame on people’s policy preferences. We found that the metaphor frames affected preferences in the pooled data, *χ* ^*2*^(4, N = 876) = 16.346, *p* = .003, Cohen’s *w* = .14. Analyzed separately, we found a marginal effect in Experiment 4 of [7], *χ* ^*2*^(4, N = 350) = 8.609, *p* = .072, Cohen’s *w* = .16, and a significant effect in our Experiment 1, *χ* ^*2*^(4, N = 526) = 13.075, *p* = .011, Cohen’s *w* = .16 (see Panels a and b of Fig 3).

To determine which response option(s) drove the omnibus effect in the pooled data, we conducted five post-hoc chi-square tests of independence (Bonferroni-corrected *α* = .01)–one for each response option–by constructing five separate a 2 (frame: *virus* or *beast*) by 2 (option chosen: yes or no) frequency tables. Table 3 shows the results of these tests and reveals that the effect was driven by the “neighborhood watches” option.

**Table 3:** Frequencies (and proportions) of selections of each policy by frame.

	Economy	Education	Patrols	Prison	Watch
Beast (n = 423)	101 (.239)	92 (.217)	120 (.284)	24 (.057)	86 (.203)
Virus (n = 453)	81 (.179)	95 (.210)	107 (.236)	27 (.060)	143 (.316)
χ^2^(1)	4.421	0.039	2.328	0.001	13.728*

**Note**. Data are pooled from Steen et al.’s Experiment 4 [7] and the current Experiment 1. Asterisk indicates significance at the Bonferroni-corrected level of .01.

That is, people who read that crime was a *virus* were more likely to endorse the proposal to “Develop neighborhood watch programs and do more community outreach,” than people who read that crime was a *beast*. Although the “neighborhood watch” option was coded as enforcement-oriented by Thibodeau and Boroditsky [5–6] and in [7], and therefore congruent with the *beast* frame, as we described above, it is, according to 2014 opinion norms, more reform-oriented and consistent with the *virus* frame in these samples.

In Thibodeau and Boroditsky’s earlier samples that included the “neighborhood watch” option (i.e. [6] Experiments 3 and 4), the overall distribution of choices (see Fig 3) differed from that of the more recent samples (see Panel c of Fig 1), *χ* ^*2*^(4, N = 1,400) = 61.281, *p* < .001. Bonferroni-corrected (*α* = .01) post-hoc tests revealed that, in the past, people were more likely to endorse policies that emphasized the economy (32.3% vs. 21.1%), *χ* ^*2*^(1) = 24.626, *p* < .001, and prison sentences (13.5% vs. 6.9%), *χ* ^*2*^(1) = 19.255, *p* < .001; people were less likely to endorse policy approaches that emphasized neighborhood watches and community outreach (15.8% vs. 26.2%), *χ* ^*2*^(1) = 21.646, *p* < .001. There was not a significant difference in the proportion of people who endorsed education (17.7% vs. 19.5%), *χ* ^*2*^(1) = .645, *p* = .422, or street patrols (20.6% vs. 26.3%), *χ* ^*2*^(1) = 6.194, *p* = .013.

The move away from policies grounded in the economy and prison system, and toward policies grounded in community outreach, suggests that there may have been a cultural shift in how people conceptualize aspects of crime (including neighborhood watch programs, prison, and policing), the economy, and education over the past six years.

To understand how these cultural shifts may have effected the interpretation of results reported in [7] as either enforcement- or reform-oriented we dichotomized the responses according to both 2011 opinion norms, and according to the more relevant 2014 opinion norms.

### Analyses based on OLD opinion norms

#### Re-analyses of Steen et al.’s experiment 4 [7]

Data from Experiment 4 of [7] was coded according to 2011 opinion norms, with the neighborhood watch option coded as enforcement-oriented and consistent with the *beast* metaphor. We reanalyzed the data according to this same coding scheme. The best-fitting logistic regression model for these data included nine parameters (main effects for frame, political ideology, age, education, sex, and interactions between the frame and age, the frame and education, and the frame and sex; AIC = 461.55; the AIC_min_ = 483.89; the AIC_max_ = 465.86).

This analysis revealed that, as reported in [7], there were no main effects of the metaphor frame (although the best fitting model included a regressor for frame it was not a significant predictor in the model). There were, however, significant effects of the covariates. Older participants were more likely to be enforcement-oriented, *B* = 0.070, *SE* = .019, *p* < .001, and people with more education were more likely to be reform-oriented, *B* = -0.513, *SE* = .141, *p* < .001. In addition, Democrats were more likely to be reform-oriented than Republicans, *B* = .848, *SE* = .848, *p* = .016 (Democrats and Independents showed similar policy preferences).

#### Experiment 1

We used the same procedure and 2011 coding scheme to analyze the data from our own Experiment 1. The best fitting model for these data included six parameters (main effects for the frame, sex, age, and political affiliation; AIC = 699.26; the AIC_min_ = 710.49; the AIC_max_ = 711.91). This model revealed significant effects of age, *B* = .022, *SE* = .010, *p* = .021, and political ideology: Republicans were more enforcement-oriented than Independents, *B* = .848, *SE* = .848, *p* = .016, and Democrats, *B* = .801, *SE* = .290, *p* = .006 (Democrats and Independents showed similar policy preferences). The frame was not discarded by the model, but it was not a significant predictor of participants’ responses.

#### Pooled data

We then pooled the data from Experiment 4 of [7] with that of our Experiment 1 (N = 876). We used the same stepwise fitting algorithm to select the best model to predict participant’s policy choice. In this case two additional parameters were included (one to test for a difference by experiment and one to test for an interaction between the experiments and frames). The best fitting model from the pooled data is shown in Table 4 (AIC = 1164.2; AIC_min_ = 565.64; AIC_max_ = 1194.6).

**Table 4:** The best fitting logistic regression model for data pooled from Steen et al.’s Experiment 4 [7] and the current Experiment 1 based on an old coding scheme.

Coefficient	*B*	*SE*	*p*
(Intercept)	-1.072	0.413	0.010
**Frame**: Virus	0.958	0.480	0.046
Age	0.043	0.011	< .001
**Pol**: Democrat	-0.232	0.152	0.127
**Pol**: Republican	0.553	0.218	0.011
Education	-0.072	0.050	0.148
Current Experiment 1	0.308	0.160	0.054
Virus * Age	-0.021	0.014	0.155

**Note.** Positively signed coefficients (*B*s) reflect an increased likelihood of selecting an enforcement-oriented response.

This model revealed a significant effect of the frame: on this coding scheme, people who read that crime was a *virus* appear more enforcement-oriented, as do older participants, and Republicans (and Republicans appear more enforcement-oriented than Democrats, *B* = .785, *SE* = .221, *p* < .001).

While some of the effects are consistent with the findings of Thibodeau and Boroditsky [5–6] (e.g., that Republicans were more enforcement-oriented), the apparent effect of the metaphor frame was exactly the reverse (i.e. the *virus* frame seemed to make people more enforcement-oriented). To examine whether this apparent discrepancy was due to an outdated coding scheme, we examined the same data but this time coding the responses according to new norming data.

### Analyses based on NEW opinion norms

#### “Watch” coded as reform-oriented

In light of the results of the current norming tasks, we recoded the neighborhood watch option as reform-oriented and consistent with the *virus* frame and then refit logistic regression models to Experiment 4 of [7] and our Experiment 1. The best fitting model for the pooled data is shown in Table 5 (AIC = 1060.9; AIC_min_ = 1096.7; AIC_max_ = 1074.0).

**Table 5:** The best fitting model of re-coded data from Steen et al.’s Experiment 4 [7] and the current Experiment 1 based on a current coding scheme.

Coefficient	*B*	*SE*	*p*
(Intercept)	-1.628	0.329	< 0.001
**Frame**: Virus	-0.474	0.231	0.041
**Sex**: Male	-0.105	0.216	0.626
Age	0.012	0.007	0.100
**Pol**: Democrat	-0.120	0.169	0.479
**Pol**: Republican	0.888	0.208	< 0.001
**Experiment**: Experiment 1	0.643	0.158	< 0.001
Virus * Male	0.478	0.304	0.116

**Note.** Positively signed coefficients (*B*s) reflect an increased likelihood of selecting the enforcement-oriented response.

This model revealed an effect of the metaphor frame in the predicted direction. People who read that crime was a *virus* were more likely to prefer a policy response that was consistent with the *virus* metaphor (i.e. to be more reform-oriented): 34.0% of participants who read that crime was a *beast* chose an enforcement-oriented response compared to 29.6% of participants who read that crime was a *virus*. Participants were 4.9 and 5.1 percentage points more likely to choose an enforcement-oriented response in Steen et al.’s Experiment 4 [7] and our Experiment 1, respectively.

In addition, Republicans were more likely to be enforcement-oriented than Independents and Democrats, *B* = 1.008, *SE* = .213, *p* < .001. On average, Republicans choose an enforcement-oriented response 50.7% of the time; Independents and Democrats choose an enforcement-oriented response 29.6% and 26.6% of the time, respectively.

Participants in the current Experiment 1 were also more enforcement-oriented than participants in Experiment 4 of [7]. However, importantly, the frame showed a similar effect in the two experiments as there was no interaction between the experiments and the frames.

Separate analyses on the data from Experiment 4 of [7] and the current Experiment 1 reveal consistent results. The best-fitting model for Experiment 4 of [7] included five predictors (frame, political ideology, time spent reading the report, and an interaction between the frame and time spent reading the report; AIC = 383.7; AIC_min_ = 387.76; the AIC_max_ = 397.0). People who read that crime was a *virus* were more likely to select a policy that was consistent with the *virus* frame (i.e. a reform-oriented response), *B* = -1.362, *SE* = .649, *p* = .036. Republicans were more likely to select an enforcement-oriented response compared to Independents, *B* = .735, *SE* = .343, *p* = .032, and Democrats, *B* = 1.053, *SE* = .355, *p* = .003.

The best fitting model for Experiment 1 included seven predictors (frame, sex, age, political ideology, and an interaction between the frame and sex; AIC = 680.57; AIC_min_ = 694.56; AIC_max_ = 692.99). People who read that crime was a *virus* were more likely to select a reform-oriented response, *B* = -.559, *SE* = .283, *p* = .048. And Republicans were more likely to prefer enforcement-oriented policies compared to Independents, *B* = .958, *SE* = .264, *p* < .001, and Democrats, *B* = 1.013, *SE* = .272, *p* < .001.

To summarize, both data sets reveal the predicted effect of metaphorical frame on policy preference, replicating prior findings [5–6]. The apparent discrepancy reported in [7] was introduced because the coding scheme in [7] relied on outdated opinion norms. While opinion on the topic of crime and people’s interpretation of different response options has shifted over time, the effect of metaphorical framing has remained.

### Experiment 2: Excluding the Neighborhood Watch Option

To further investigate the reliability of the framing effect and to ensure that it could be elicited in the absence of the relatively ambiguous “neighborhood watch” option, we conducted a follow-up experiment in which participants were asked to choose between two policy approaches. In Experiment 2 there were two framing conditions (*virus* and *beast*) and two response options: “education” and “patrols”. We chose these two policy options because they clearly differed along the two relevant dimensions: the “patrols” option was viewed as more consistent with the *beast* frame and was rated as more enforcement-oriented; the “education” option was viewed as more consistent with the *virus* frame and was rated as more reform-oriented. In addition, both of these response options were widely endorsed in previous experiments, unlike, for instance, the “prison” option, which was viewed as effective by only a small proportion of participants.

In this case, we found that 54.4% of policy preferences were congruent with the metaphor frame (i.e. on coding the “patrols” option as congruent with the *beast* metaphor and the “education” option as congruent with the *virus*), *χ* ^*2*^(1, N = 521) = 4.240, *p* = .039, *w* = .09. Republicans were more enforcement-oriented than Democrats, *B* = .921, *SE* = .282, *p* = .001, and Independents were more enforcement-oriented than Democrats, *B* = .538, *SE* = .197, *p* = .006 (the best fitting model included seven parameters: frame, sex, political ideology, time spent reading the report, and an interaction between the frame and sex; AIC = 702.49; AIC_min_ = 712.23; AIC_max_ = 713.98).

That is, 61.0% of participants who read that crime was a *beast* preferred a policy that emphasized increasing “patrols” over reforming “education.” In contrast, 53.4% of participants who read that crime was a *virus* preferred the enforcement-oriented policy. For context, 70.7% of Republicans, 61.1% of Independents, and 48.8% of Democrats preferred the enforcement-oriented approach.

### Covertness

Finally, we wished to test for evidence that the influence of the frame was due to participant’s active use of the metaphors to reason about crime in all three experiments. Consistent with [6], we reasoned that if people were actively using the frame to make their policy preference, they should be able to remember the frame a few moments later.

To test whether people actively used the metaphor to influence their reasoning, we analyzed data from the follow-up cued recall question. In Steen et al.’s Experiment 4 [7] and the current experiments people were asked whether they could remember the metaphor that was used to frame the issue in the original version of the crime report. We pooled data from these three experiments (from conditions in which a metaphor frame and the original crime report were used) and fit an initial model without the recall information (N = 1,123). This model included the full set of predictor variables (main effects for frame, age, sex, political ideology, education, and time spent reading the report, as well as a categorical predictor to account for differences in enforcement-orientation between the experiments; and interactions between the frame and each of the other variables).

The best fitting model included two predictors (five parameters)–frame and experiment (AIC = 1,440.6; AIC_min_ = 1509.3; AIC_max_ = 1456.5). Critically, the model revealed that people who read the crime was a *beast* were more likely to select an enforcement-oriented policy response, *B* = 1.435, *SE* = .602, *p* = .017.

In each sample, a majority of participants (64.6% overall) were able to recall the frame (the exact word or a close synonym), *χ* ^*2*^(1, N = 1,123) = 95.22, *p* < .001, Cohen’s *w* = .29. However, there were a substantial number in each sample who did not remember the frame (41.7%, 35.6%, and 28.3% Steen et al.’s Experiment 4 [7] and the current Experiments 1 and 2, respectively). We found that people were more likely to remember the *virus* (70.2%) than the *beast* frame (58.3%), *χ* ^*2*^(1, N = 1,123) = 19.287, *p* < .001, Cohen’s *w* = .13.

However, we did not find that people who remembered the frame were more likely to choose a consistent policy response. Adding this measure to the previously reported model (as an interaction with the frame) revealed that participants in the *beast* condition who remembered the frame were no more enforcement- (or reform-) oriented than people who did not, *B* = .078, *SE* = .179, *p* = .661, nor were participants in the *virus* condition who remembered the frame more reform- (or enforcement-) oriented, *B* = .118, *SE* = .201, *p* = .557 (AIC = 1,444.1). We also found that when we included this measure in a full model, the stepwise selection algorithm did not include it among the best-fitting subset of predictors (AIC_max_ = 1460.1).

That is, consistent with earlier findings [6] we found that people who did not remember the metaphor frame were just as likely to show an influence of the frame. This suggests that the metaphor covertly affects the way at least some people reason about crime.

### Summary

The analysis of Steen et al.’s Experiment 4 [7] and our Experiments 1 and 2, in which a metaphor frame (*virus* or *beast*) was used in conjunction with the original crime report, reveal a reliable effect of the frame on people’s policy preference. The apparent discrepancy reported in [7] was introduced because the coding scheme in [7] relied on outdated opinion norms. In the 2014 sociopolitical context “Develop[ing] neighborhood watch programs and do[ing] more community outreach” was viewed as more reform-oriented and consistent with the *virus* frame. Coding the data according to current opinion norms reveals a consistent effect of metaphorical frame.

In addition, we found an effect of the frames on raw (i.e. uncoded) policy preferences in the experiments that included five response options. In particular, people who read that crime was a *virus* were more likely to endorse the “neighborhood watch” option compared to people who read that crime was a *beast*. In Experiment 2, we found that the metaphor framing effect did not rely on the presence of this response option. When people were asked to choose between two options that clearly differed the degree to which they were reform- or enforcement-oriented (and consistent with the *virus* and *beast* metaphors), we found a reliable effect of the metaphor frames.

We also consistently found that Republicans were more likely to endorse enforcement-oriented policy approaches (regardless of how the neighborhood watch option was coded or how many policy options were included).

For an ecological context, we can compare the shift associated with changing the metaphor to predictable differences that we found by political affiliation. Whereas Republicans’ likelihood of selecting an enforcement-oriented response was 10–20 percentage points greater than that of Independents or Democrats, people who read that crime was a *beast* were 5–7 percentage points more likely to choose an enforcement-oriented response. Of course, the size of the shift in opinion induced by metaphors in these studies should be interpreted in context. The materials were designed to test whether a change of just one metaphorical word could create a difference in how people understood and reasoned about the problem of crime. The materials were not designed to maximize differences. In natural contexts, metaphors can be extended, repeated, and amplified with supporting information, and so may lead to larger shifts in opinion.

Further, we found evidence that the metaphor covertly influenced at least some people’s reasoning about crime, as the effect of the frame was not restricted to people who remembered the frame.

One additional conclusion that can be drawn from these experiments is that it can be difficult to study framing in a dynamic real-world context. The issue of crime is consistently a focal issue in the media, although the way it is covered changes in predictable and unpredictable ways depending on the media outlet and as a result of salient events and political cycles [12–14]. These variables can have profound effects on the way people think about crime [15–18]. As a result, it is wise to use within-sample norming studies to gauge the degree to which people view response options as consistent with frames.

### Can we tell which of the two metaphors (or both) are doing the work?

The results presented above and in prior work show that framing crime as a *beast* makes people more likely to prefer enforcement-oriented solutions than if crime had been framed as a *virus*. The results so far inform us that there is a psychological difference between the two metaphorical frames. However they do not tell us how the metaphors are shaping people’s views relative to what they would have thought without a metaphor. Are both metaphors equally contributing to the separation of opinion in the two conditions, or is just one of the metaphors doing all the work? It is also possible, for example, that both *beast* and *virus* metaphors make people more enforcement-oriented than they would have been without the metaphors, but the effect of the *beast* metaphor is stronger. Likewise, it is possible that both the *beast* and *virus* metaphors make people more reform-oriented than they would have been without the metaphors, but the effect of the *virus* metaphor is stronger.

These questions raise an interesting conundrum. What is an appropriate baseline that we can use to measure against? Here, we consider several possibilities:

#### Compare to opinion prior to study

One might try to obtain a baseline by asking people’s opinions on the topic prior to the study (without reading about the crime situation in Addison, as in Experiments 1 and 2 of [7]). There are several problems with this approach. First, reading about a specific instance of crime in a city, and learning the specific numbers of offenses, may in itself change what people imagine when they approach thinking about crime. As a result, any difference of opinion observed between measures taken before reading the paragraph and measures taken after reading the paragraph may be due to the information and contextual cuing contained in the paragraph itself (regardless of metaphors). Second, if pre- and post-test measures are taken close to each other in time, with participants being asked the same questions twice, the design becomes vulnerable to strategic decisions on the part of the participants.

For some, the pre-test may serve as an anchor, as people tend to prefer consistency in expressing their attitudes and preferences (e.g., [19]). Others may think that they are *supposed* to change their preference–or else why would they be asked this question a second time? In such cases participants may wish to please the experimenter and try to guess how the experimenter would want their answer to change from pre-test to post-test.

One fix might be to give the pre-test a year or a few months before the post-test so that people have a chance to forget about their initial judgment. This method however would still be susceptible to the possibility that just reading the paragraph about crime in itself (regardless of metaphors) shifts people’s thinking about crime in the moment.

Further, while it might be intuitively tempting to think that a pre-test is needed in order to know whether the metaphorical frames really caused a change in people’s preferences, this intuition stems from an under-appreciation of the logic of random assignment to conditions in experimental design.

Experimental designs that are fully between-subjects are frequently used to test the effects of interventions on behavior. A basic premise of experimental design is that by randomly assigning experimental subjects to different treatment conditions, one can infer that differences in outcomes must be attributed to differences in treatment. The logic of random assignment allows one to infer a causal link between the treatment variable and the outcome measure. The same random assignment logic applies whether the subjects of study are pea plants and the treatment conditions vary soil moisture, or the subjects of study are people and the treatment conditions are different linguistic frames for a problem. Consider Kahneman and Tversky’s [20] seminal work on gain versus loss framing. As a result of these kinds of experiments, it is widely accepted that people are more likely to prefer a sure gain but a probabilistic loss, despite the fact that participants were not pretested for their willingness to take probabilistic risks.

#### Compare to a “neutral” or “non-metaphorical” framing

Another approach to obtaining a baseline against which to measure the effects of the two metaphors is to run a “neutral” or a “non-metaphorical” condition. This path too is problematic. How would one establish that any alternative framing is indeed “neutral” with respect to the two metaphorical framings?

For example, suppose we replace “Crime is a beast/virus ravaging the city of Addison” with “Crime is a problem ravaging the city of Addison” (as in [7]). There are two concerns.

1. How do we know that *problem* is not in fact more similar to one of the metaphors than the other? For example, if we were to find that responses to the *beast* frame are the same as to the *problem* frame, are we licensed to conclude that it is the *virus* frame that affects people’s thinking? Clearly not. All we would know is that the *beast* frame and the *problem* frame are more similar to each other than the *virus* frame. Since there is no external standard by which we could judge the *problem* frame as neutral, comparing the other two conditions to the *problem* frame doesn’t get us any closer to answering which of the frames may or may not have more of an effect on reasoning.
2. Linguistic stimuli are complex and people’s responses to linguistic stimuli depend strongly on many properties such as frequency, vividness, conventionality, emotional valence, arousal and so on. Replacing *beast* or *virus* with *problem* introduces a host of uncontrolled changes along these dimensions. The same issue holds if one simply removes the words *beast* or *virus* so that the report begins, “Crime is ravaging the city of Addison.” Replacing or removing the key metaphorical words does not make a neutral stimulus, it makes a *different* stimulus.

In the following section we report data from a norming task (Norming Study 2; Norming Task 3) in which we quantify the degree to which several frames for crime are emotionally salient, metaphorical, and conventional. These norming data illustrate several basic dimensions on which “crime is a problem” is a not an equivalent framing to “crime is a beast/virus.”

### Norming the Frames

We sought to quantify four “crime is a …” statements along several relevant dimensions (see Table 6). The four frames included: *beast*, *virus*, *problem*, and *horrific problem*.

**Table 6:** Mean ratings of three “Crime is a …” frames (*sd*).

	Ranked Severity	Rated Severity	Metaphoricity	Conventionality	Preferred
*Beast*	2.23 (.987)	79.6 (17.0)	88.7 (13.9)	33.4 (24.1)	35 (14.1%)
*Virus*	2.37 (.916)	78.0 (15.9)	86.6 (16.0)	40.1 (26.1)	40 (16.1%)
*Problem*	3.55 (.916)	58.1 (26.8)	22.2 (25.5)	88.4 (15.3)	59 (23.8%)
*Horrific*	1.94 (.911)	81.3 (16.7)	29.2 (26.8)	73.3 (15.3)	114 (46.0%)

In this norming study participants first rank-ordered the four frames according to how severe the classifications made the crime problem seem. A repeated measures ANOVA revealed that there were differences between the frames on this measure, *F*[3, 741] = 109.08, *p* < .001. Bonferroni-corrected post-hoc paired t-tests (*α* = .008) revealed that the *problem* frame connoted a less severe instance of crime than any of the others (*p*s < .001). In contrast, the *horrific problem* frame was viewed as more severe than the *beast*, *t*[247] = 2.834, *p* = .005, or *virus* frames, *t*[247] = 3.333, *p* = .001. No other comparisons were significant.

Second, participants rated the frames according to how severe they made crime seem. This measure yielded similar results, *F*[3, 741] = 109.80, *p* < .001. In this case Bonferroni-corrected post-hoc paired t-tests (*α* = .008) revealed that the *problem* frame connoted a less severe instance of crime than any of the others (*p*s < .001). But this measure did not reveal differences between any of the other pairs of frames.

Third, participants rated how metaphorical the frames seemed, *F*[3, 741] = 778.2, *p* < .001. Paired t-tests revealed differences between each pair of frames (*p*s < .001; Bonferroni-corrected) except *beast* and *virus*, which were viewed as similarly metaphorical.

Fourth, participants rated how common or conventional the frames seemed, which also yielded significant differences, *F*[3, 741] = 389.41, *p* < .001, in this case between each pair of frames (*p*s < .001; again, Bonferroni-corrected).

Finally, we measured participants’ preference for the different frames. Participants were asked which frame they would prefer in order to convey a severe instance of crime, *χ* ^*2*^(3, N = 248) = 63.323, *p* < .001, *w* = .50. The most popular frame was *horrific problem* (46.0%), followed by *problem* (23.8%), *virus* (16.1%) and *beast* (14.1%).

These results suggest that a *problem* frame is not only less metaphorical than the *virus* or *beast* frames, but also is perceived to be describing a less severe instance of crime. In addition, the *problem* frame was viewed as a more conventional way of describing crime and participants preferred it over the metaphorical expressions.

The *horrific problem* frame was a better match to the *virus* and *beast* frames in some ways (i.e. it seemed to convey a similarly severe instance of crime.) However, like the *problem* frame it was viewed as a more conventional way of talking about crime and was judged to be more preferable than either of the metaphor frames.

Based on these results, we can be confident that differences in participants’ policy preferences elicited by the *virus* and *beast* frames do not stem from differences in the degree to which the frames are metaphorical or connote a severe instance of crime. However, a *problem* frame (or even a *horrific problem* frame) fails to control for important linguistic variables. As a result, neither of these “non-metaphorical” frames are “neutral” in the desired sense: neither differs from the metaphorical frames solely along the dimension of metaphoricity.

Further, it may not be possible to boil the differences between the frames down into one or two underlying dimensions as both metaphor frames seem to instantiate knowledge structures that dynamically affect the way that people build representations of the crime problem that is described in the report [5–6].

## Discussion

In this paper, we have considered some of the factors that influence a metaphor framing effect for an important real world issue. Although the overall landscape of opinion on the topic of crime has shifted, we find that metaphors for crime continue to have an influence on people’s reasoning about crime.

The findings to date also shed some light on the mechanisms through which metaphors may influence thinking. Both prior and current work finds that changing a single metaphorical noun is sufficient to initiate a shift in thinking. However, how people encounter this noun is important. Our prior work shows that simply thinking about the disconnected word “beast” or “virus” separate from the narrative about crime does not create a shift in opinion. The noun does not act alone. When a noun like beast or virus is used as a metaphor for crime, it instantiates a knowledge frame that then can coerce other information about the problem into the frame. We crafted our experimental materials to contain many elements that could be interpreted differently if one were thinking of them in the context of beasts as opposed to viruses. For instance, embedded in the description of crime are words and phrases–like “in good shape” and “no obvious vulnerabilities.” These may take on different meanings when preceded by the *virus* or *beast* frame.

This interpretation is supported by the finding that the metaphorical frame is most powerful if it is encountered early. This way the frame can actively shape the process of building a representation for the problem. Indeed, we have found that when people encounter the metaphorical word at the end of the description, rather than the beginning, the metaphor does not influence their crime-reduction policy preferences [5]. This finding is also consistent with prior work on meaning instantiation, which has shown that people are better able to organize and remember descriptions of events and procedures when the topic of the description is presented early on (e.g., people are better able to understand a procedural description of washing clothes when they know they are reading a procedural description of washing clothes [21]).

Our investigations also offer a number of lessons for the burgeoning culture of rapid direct replication in psychological science. First, this work clearly demonstrates the advantages of publicly sharing data and materials. The fact that data from [7] were available for re-analysis allowed us to discover an interesting new pattern and to follow up on this discovery with our own studies.

Second, this example highlights the importance of thinking about replication not in terms of individual studies, but in terms of lines of investigation. Often the interpretation of the results of any one experiment depends on many other ancillary pieces of data, norming results, and control conditions reported elsewhere in the same paper or in the same line work more broadly. In this case, arriving at a proper interpretation of results depended on having up-to-date opinion norms. Arriving at a meaningful culture of replication will require going beyond a focus on direct replication of disconnected single studies, and instead shifting to a theoretically-informed consideration of the broader set of dependencies needed for interpreting any given finding.

Third, this work highlights the importance of analyzing the full scope of collected data. For instance, although Steen et al. [7] did not find evidence of a metaphor framing when the five response options were dichotomously coded, such a pattern is evident in the, raw, uncoded data.

### Conclusion

With three norming tasks and two experiments, we further explored a metaphor framing effect in an important and dynamic real world context: crime. The three norming tasks highlight critical factors for researchers to keep in mind when conducting framing studies in real world contexts. The first two reveal the importance of using up-to-date norming data; the third illustrates limitations of comparing metaphorical to non-metaphorical frames.

The two experiments replicate prior work [5–7] and pinpoint the cause of a reported null effect [7]. The first experiment shows that people are more likely to pursue enforcement-oriented policy interventions when crime is framed as a *beast* compared to when crime is framed as a *virus*. However, this experiment included a relatively ambiguous policy option–“neighborhood watches.” In a follow-up experiment that excluded this option, we again found a reliable metaphor framing effect, showing that the effect of the metaphors did not depend on the presence of this ambiguous policy response.

In sum, the results confirm that natural language metaphors can affect the way we reason about complex problems.
